# Prediction and characterization of protein-protein interaction networks in swine

**DOI:** 10.1186/1477-5956-10-2

**Published:** 2012-01-10

**Authors:** Fen Wang, Min Liu, Baoxing Song, Dengyun Li, Huimin Pei, Yang Guo, Jingfei Huang, Deli Zhang

**Affiliations:** 1 College of Life Science, Center for Bioinformatics, Northwest A&F University, Yangling, Shaanxi 712100, China; 2 College of Veterinary Medicine, Northwest A&F University, Yangling, Shaanxi 712100, China; 3 College of forestry, Northwest A&F University, Yangling, Shaanxi 712100, China; 4 State Key Laboratory of Genetic Resources and Evolution, Kunming Institute of Zoology, Chinese Academy of Sciences, Kunming, Yunnan, P.R. China

**Keywords:** protein-protein interaction network, Interolog, D-MIST, M-MIST topological properties, Pfam domain annotations, GO annotations

## Abstract

**Background:**

Studying the large-scale protein-protein interaction (PPI) network is important in understanding biological processes. The current research presents the first PPI map of swine, which aims to give new insights into understanding their biological processes.

**Results:**

We used three methods, Interolog-based prediction of porcine PPI network, domain-motif interactions from structural topology-based prediction of porcine PPI network and motif-motif interactions from structural topology-based prediction of porcine PPI network, to predict porcine protein interactions among 25,767 porcine proteins. We predicted 20,213, 331,484, and 218,705 porcine PPIs respectively, merged the three results into 567,441 PPIs, constructed four PPI networks, and analyzed the topological properties of the porcine PPI networks. Our predictions were validated with Pfam domain annotations and GO annotations. Averages of 70, 10,495, and 863 interactions were related to the Pfam domain-interacting pairs in iPfam database. For comparison, randomized networks were generated, and averages of only 4.24, 66.79, and 44.26 interactions were associated with Pfam domain-interacting pairs in iPfam database. In GO annotations, we found 52.68%, 75.54%, 27.20% of the predicted PPIs sharing GO terms respectively. However, the number of PPI pairs sharing GO terms in the 10,000 randomized networks reached 52.68%, 75.54%, 27.20% is 0. Finally, we determined the accuracy and precision of the methods. The methods yielded accuracies of 0.92, 0.53, and 0.50 at precisions of about 0.93, 0.74, and 0.75, respectively.

**Conclusion:**

The results reveal that the predicted PPI networks are considerably reliable. The present research is an important pioneering work on protein function research. The porcine PPI data set, the confidence score of each interaction and a list of related data are available at (http://pppid.biositemap.com/).

## 1 Background

Protein-protein interactions (PPIs) [1] were previously determined based on only a single molecule, thus a comprehensive understanding of the entire biological processes could not be acquired. To obtain a thorough perspective, merely listing the proteins of an organism is far from enough: all the interactions among them need to be delineated as well [1]. The investigation of these processes demands the utilization of proteome-wide PPIs, and constructing a PPI network can lead to a more complete understanding of biological processes. A crucial step toward this feat is a complete and accurate mapping of the networks of physical DNA and RNA interactions and PPIs, the "interactome network" of an organism [2]. The yeast *Saccharomyces cerevisiae *has been used to develop a eukaryotic unicellular interactome map [3-6]. The current research aims to decipher the porcine network of proteome PPIs by constructing of a porcine PPI network using three methods. The experimental techniques for the detection and validation of PPIs are time-consuming [7], and labor-intensive, and these experimentally detected interactions show high false negative [8] and positive rates [7,9,10]. In the present paper, we used three computational approaches to predict porcine PPIs and validated our predictions. These methods are based on the Interolog [11], domain-motif interactions from structural topology (D-MIST) [12] and motif-motif interactions from structural topology (M-MIST). We also described in detail the methods for PPI network visualization and analysis [13]. Accession to PPI information will greatly aid biological research and potentially make discovery of novel drug targets much easier [13].

The Interolog approach, a method presented several years ago, focuses on the building of PPI maps. The main idea behind this method is the transfer of known interactions from model organisms to other species based on the predicted orthology of the respective proteins [14]. Thus, if the interolog of a protein interaction exists in many other organisms, this protein interaction will score highly [7].

D-MIST is based on a two-step approach. First, potential domain-binding motifs are extracted from structural data. These motifs are then converted to sequence profiles in the form of position-specific scoring matrices (PSSMs) [12]. If one protein has a domain and another has corresponding motif information, the two proteins are considered to interact with each other.

M-MIST method is based on motif-motif contacts derived from PPIs from the Biomolecular Interaction Network Database (BIND) [15]. If a motif group pair is found in the observed PPIs, other protein pair matches with the motif group pair, then these two proteins can be thought to have interaction.

There are many well-known databases about human PPIs, such as DIP [16], HPRD [17] and MINT [18], which include 3,376, 39,194, and 22,677 human PPIs, respectively. However, no data of pig are available from them. IntAct [19], BIND [15], Biogrid [20], MIPS [21], STRING [14], and other databases also include information of human PPIs, but seldom of pig. Thus, the nature of the mediation of swine PPIs by molecular mechanisms, the heart of almost every biological process, remains unclear.

The existing methods that can be used to predict PPIs include Interolog [11], D-MIST, subcellular localization [22], Bayesian networks [23], phylogenetic profiles [24,25], network integration, literature mining method, preferential attachment rule[26], duplication and divergence rule [26] and others. While all of these approaches can be used for interaction prediction, their aims are different. Interolog is the primary method widely used and proved reliable for predicting the PPIs of model organisms [27]. In the PPI network, nodes are generally used to represent proteins and edges are used to represent interactions [28], if interactions exist between proteins. In this work, we generated porcine PPI maps, which can provide new insights into the protein function research.

## 2 Results

**Figure 1:**
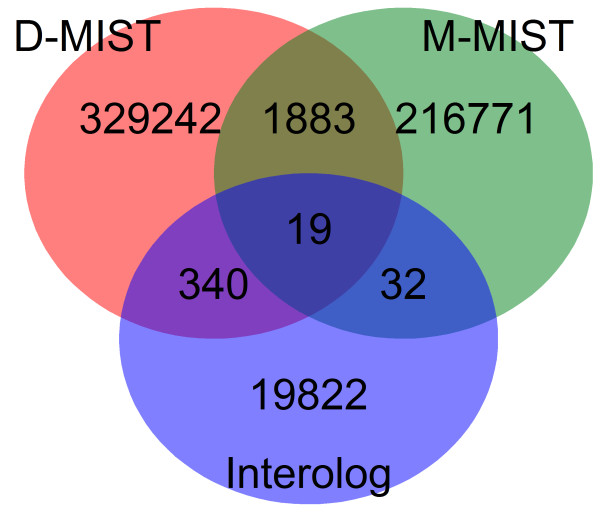
**The number of overlapping PPIs of the three methods**. The D-MIST, M-MIST, and Interolog methods complement each other, as they operate on fairly disjointed sets.

We predicted a total of 567,441 porcine PPIs using 3 methods and constructed 4 PPI networks: Interolog, D-MIST, M-MIST, and a combination of the 3 networks. Table 1 presented the three approaches used for the analysis of porcine PPI data. The PPIs under the three methods could lead to many local perturbations in the network, and the global properties of the four networks are not likely to change significantly (Table 2). The overlap of the interactions among the three methods was shown in Figure 1.

**Table 1:** The number of predicted protein-protein interactions

Method	Predicted PPI
Interolog	20,213
D-MIST	331,484
M-MIST	218,705

**Table 2:** Global properties of the four networks

Property	Interolog	D-MIST	M-MIST	Merged network
Nodes	5,726	6,163	3,873	11,955
Edges	20,213	331,484	218,705	567,441
Clustering coefficient	0.105	0.267	0.123	0.223
Network diameter	13	9	9	11
Network radius	1	1	1	1
Shortest paths	94%	98%	96%	98%
Characteristic path length	4.148	3.530	3.445	3.554

**Figure 2:**
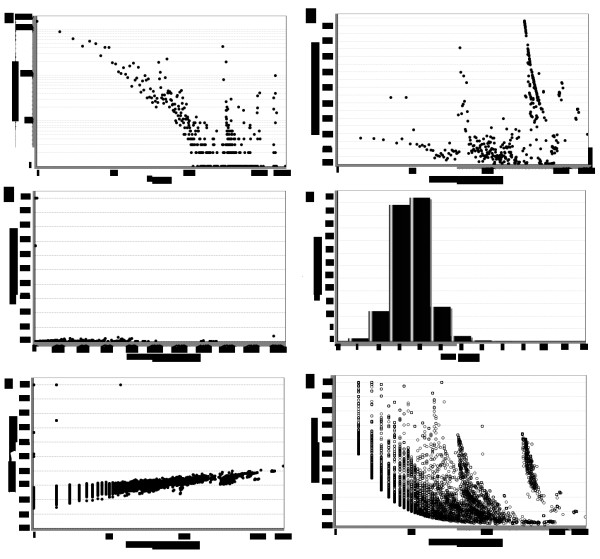
**The properties of the merged network**. (a) Degree distribution. The connectivity distribution of the proteins obeys the power-law distribution, containing many proteins with a few interactions and a limited number of proteins with a large number of interactions. (b) Betweenness centrality. (c) Closeness centrality. (d) Clustering coefficient. (e) Shortest path. (f) Topological coefficient.

The degree of a node corresponds to the number of interactions it has with the neighboring molecules [29]. Highly connected proteins (hubs) with central roles in the network architecture are more essential in the PPI network than proteins with only a small number of interactions [30]. In the current research, using *k *to represent degree, and *n_k _*to represent the number of nodes of the degree *k *(Additional file 1), we analyzed the degrees of the nodes (Additional file 2), and found that the degree distributions (Figure 2a) in the fourth network obeyed the power-law. The probability *P*(*k*) of nodes was *P*(*k*)≈k^-1.004^, *R^2 ^*= 0.559. This finding suggested that the network contained a small number of highly connected proteins and that a large number of proteins possessed only a few connections. In biological networks, this phenomenon is the so-called scale-free property. The scale-free nature of a protein interaction network indicates that a limited number of proteins have a large number of interactions [31]. Highly connected proteins are more important for fitness than less-connected proteins because randomly removing these proteins would likely result in fitness defect. The network is highly tolerant of the random removal of a protein, but vulnerable to the targeted removal of hub proteins, whose removal drastically changes the network topology [30]. The scale-free property also indicates resistance to random node failure [32]. The betweenness centrality (Figure 2b) [33]*C_b_(n) *of a node *n *was calculated by *C_b_*(*n*) = ∑*_s≠n≠t _*(*σ_st _*(*n*)/*σ_st_*), where *s *and *t *were nodes in the network different from *n, σ_st _*denoted the number of the shortest paths from *s *to *t*, and *σ_st _*(*n*) was the number of the shortest paths from *s *to *t *that *n *laid on. The betweenness value for each node *n *was normalized by dividing by the number of node pairs excluding *n*: (*N*-1) (*N*-2)*/2*, where *N *was the total number of nodes in the connected component that *n *belonged to. Thus, the betweenness centrality of each node is a number between 0 and 1. The closeness centrality (Figure 2c) [34]*C_c_(n) *of a node *n *meant the reciprocal of the average shortest path length and was calculated by *C_c_*(*n*) = *1*/*avg*(*L*(*n*,*m*)), where *L*(*n*,*m*) was the length of the shortest path between the nodes *n *and *m*. The closeness centrality of each node was a number between 0 and 1. In undirected networks, the clustering coefficient (Figure 2d) *Cn *of a node *n *was calculated by *C_n _*= 2*e_n_*/(*k_n_*(*k_n_*-1)), where *k_n _*is the number of neighbors of *n *and *e_n _*is the number of connected pairs between all neighbors of *n *[35,36]. The length of the shortest path (Figure 2e) between the nodes *n *and *m *was *L *(*n*,*m*). The shortest path length distribution gave the number of node pairs (*n*,*m*) with *L*(*n*,*m*) = *k *for *k = 1,2,..*.. The topological coefficient (Figure 2f) [37]*T_n _*of a node *n *with *k_n _*neighbors was calculated by *T_n _= avg *(J (*n*,*m*))/*k_n_. J*(*n*,*m*) means all nodes *m *that share at least one neighbor with *n*. The value *J *(*n*,*m*) was the number of neighbors shared between the nodes *n *and *m*, plus one if there was a direct link between *n *and *m*. The diameters of the four networks imply the small-word property (Table 2). Other properties, such as average clustering coefficient, network radius, shortest path, characteristic path length, number of nodes, and number of edges, were presented in Table 2. We did not find any significant difference in the global network properties of the four networks.

Using the Interolog method to predict the orthologs of human, mouse and rat with those of pig, we got 20,213 interactions out of 25,767 porcine proteins, and 70 (Additional file 3) interactions associated with Pfam domain-interacting pairs in the iPfam database were verified by applying the Pfam domain annotation method. In the Pfam domain annotations, by setting an e-value cutoff at 0.01, 4,675 proteins could be assigned to Pfam domain annotations, constructing 19,712 PPIs. For comparison, we randomly chose 20,213 pairs of porcine protein from all pig proteins every time using sampling with replacement, and we preformed this process for 100 times altogether. However, in the 100 randomized networks, an average of only 4.24 interactions was associated with Pfam domain-interacting pairs in the iPfam database, (*p*-value = 0.000) (Table 3). In GO annotations, we considered that the two proteins interacting with each other if they shared at least one GO term in any of the three GO categories [38], and we found 52.68% of the predicted PPIs sharing GO terms. For comparison, 10,000 randomized PPI networks were constructed. The results showed that the number of PPI pairs sharing GO terms in the 10,000 randomized networks reached 52.68% is 0 (Additional file 4), suggesting that the predicted PPI networks have high statistical significance (*p*-value = 0.000). This method achieved an accuracy rate of about 0.92 at a precision of about 0.93 (Table 4), emphasizing that the Interolog method was helpful in the accurate and precise prediction of porcine PPIs.

**Table 3:** The Pfam domain annotations

	associated with pfam domains		
		
method	prediction	random	p-value
Interolog	70	4.24	0.000
D-MIST	10,495	66.79	0.000
M-MIST	863	44.26	0.000

**Table 4:** The accuracy and precision of the three methods

Method	accuracy	precision
Interolog	92.20%	92.97%
D-MIST	53.15%	73.64%
M-MIST	50.1%	75.03%

The D-MIST method is based on PSSMs, an interaction predicted between proteins containing interacting domains and proteins with one or more of the interacting profiles associated with those domains [12]. Using this method, 331,484 interactions were predicted, and 10,495 (Additional file 3) interactions associated with Pfam domain-interacting pairs in the iPfam database were verified using the Pfam domain annotation method. Using a 0.01 e-value cutoff, 5,515 proteins could be assigned Pfam domain annotations, constructing 330,054 PPIs. To facilitate comparison, 331,484 pairs of porcine protein were randomly selected from all pig proteins every time using sampling with replacement, and we conducted this process for a total of 100 times, and an average of only 66.79 interactions was associated with Pfam domain-interacting pairs in the iPfam database (*p*-value = 0.000) (Table 3). In GO annotations, it showed 75.54% of the predicted PPIs sharing GO terms. It was found that the percentage of PPI pairs sharing GO terms in the predicted PPI network was consistently higher than the largest percentage in the 10,000 randomized networks, suggesting that the predicted PPI networks have high statistical significance (*p*-value = 0.000) (Additional file 4). When assessing the quality of interaction data, accuracy and precision need to be considered[9]. This method yielded an accuracy of 0.57 and a precision of 0.74 (Table 4).

Using more than 10,000 structural PPIs, we identified the motifs in the binding sites and extracted them from BIND. The interacting residues were defined as polypeptide segments of five residues or longer, in which the amino acid side chains were < 4 Å from the interacting proteins [12]. Based on this idea, 218,705 interactions were predicted, and 863 (Additional file 3) interactions associated with Pfam domain-interacting pairs in the iPfam database were verified using the Pfam domain annotation method. At a cutoff e-value of 0.01, our predictions yielded 3,384 proteins for Pfam domain annotations, constructing 217,983 PPIs. We also calculated the randomized PPIs to compare them with the prediction using annotated proteins, and 218,705 pairs of porcine protein were randomly extracted from all pig proteins every time using sampling with replacement, and this process was repeated 100 times, an average of only 44.26 interactions was associated with Pfam domain-interacting pairs in the iPfam database (*p*-value = 0.000) (Table 3). In GO annotations, we calculated 27.20% of the predicted PPIs sharing GO terms, however, in the 10,000 randomized networks, none of them was achieved 27.20%, indicating the predicted PPI networks has high reliability. (*p*-value = 0.000) (Additional file 4). Accuracy and precision were also tested to assess the predictions in this method. An accuracy of about 0.50 and a precision of about 0.75 were achieved (Table 4), indicating that the discriminative power of the method.

We merged the results of the three methods using cytoscape, and a total of 567,441 PPIs were obtained, and the lowest accuracy rate was greater than 50%, and the coverage of the three results are all 100%. In Figure 2, the topological properties of the merged network were visually presented. For the network, the average number of neighbors was the average degree of a node in the network. The porcine PPI data set, the confidence score of each interaction and a list of related data were available at (http://pppid.biositemap.com/).

## 3 Discussions

In the current work, we conducted a comprehensive prediction of porcine PPI inferred from three methods. We studied PPI networks, including Interolog, D-MIST, M-MIST, and a combination of the three. All the four networks were significantly more accurate than we expected. However, the results obtained using the three methods did not match well and showed only small overlaps. The production of this result may be due to that the three methods have different emphasis: Interolog is focus on similarity between sequences; D-MIST emphasizes similarity between domains and similarity between motifs; M-MIST underlines similarity between motifs. The number of overlapping PPIs between D-MIST and M-MIST was 1,902, that between D-MIST and Interolog was 359, and that between M-MIST and Interolog was 51. After verification, each method has certain accuracy. Therefore, the three methods complement each other, and thus provide preliminary reference for related analysis. This finding showed the complexity and diversity of the PPIs, and that the methods have inherently low reproducibility and may not affect some of the interactions. Therefore, for large-scale PPIs studies, combination of these different methods could yield more abundant and accurate results.

Comprehensive analysis of the porcine proteome presents an extraordinary challenge. A powerful first step towards addressing this challenge is to develop proteome-scale interaction maps and a framework upon which a complete understanding of biological processes can be obtained. The three methods achieved accuracies of about 0.92, 0.53, and 0.50 and precisions of about 0.93, 0.74, and 0.75, respectively. The Interolog method had the highest accuracy, whereas the two other methods had similar accuracies, exhibiting the reliability of the M-MIST method.

Even the most reliable techniques could produce a large number of false-positives, so the three approaches we used would inevitably produce a considerable number of false-positives. These methods suffer from information shortage on time and space. Each of the three methods for identifying porcine PPIs has its own weak points. The Interolog method has high accuracy, but it is only applicable to human, mouse and rat. Higher accuracy rates may be achieved by increasing the number of species used in the method. In D-MIST method, the number of species (204) is sufficient. However, in spite of the presence of PSSM, the analysis only relates to domains with five or more putative interactors. Therefore, domains not frequently found in the set of protein interactions are excluded [12]. In M-MIST method, the difficulties encountered are overcome by establishing interaction maps using about 730 species and viruses. As long as there is at least one MOTIF interactor, it could be retained and used to establish the interaction maps. The disadvantage of this method is that a large number of resulting species are not carefully selected. Thus, the accuracy of this method is similar to that of the D-MIST method and does not increase.

We used the iPfam database and GO annotations to assess the reliability of the predicted PPIs. The results showed that 70, 10,495, and 863 interactions were related to Pfam domain-interacting pairs in the iPfam database, whereas an average of only 4.24, 66.79, and 44.26 randomized interactions were related to Pfam domain-interacting pairs in the iPfam database. And on GO annotations, it showed that 52.68%, 75.54%, 27.20% of the predicted PPIs sharing GO terms in the three methods respectively, and that the percentage of PPI pairs sharing GO terms in the predicted PPI network was far higher than the percentage in the 10,000 randomized networks, suggesting that the predicted PPI networks have high statistical significance (Additional file 4).

Most of the porcine protein data have not been certificated experimentally, which may be one of the reasons for the low accuracy of our predictions. And the predicted network is expected to become more reliable with the increasing quantities of porcine proteins.

**Figure 3:**
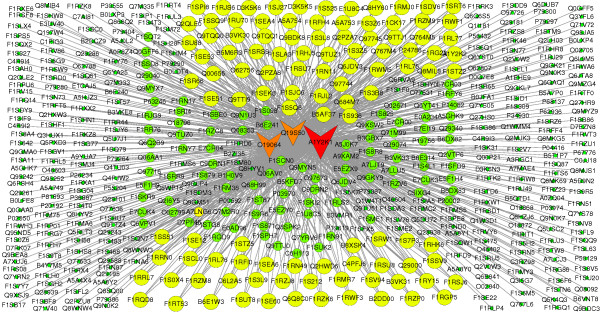
**Part of the hub nodes of the merged network**. The nodes represented by circles and V are proteins and the V nodes represent the three hub nodes A1Y2K1, O19064, and Q19S50. If the three hub nodes are removed, the network will be damaged.

One of the main applications of the PPI network is the prediction of protein functions. In the current research, protein functions were inferred based on their connections in the network [39]. The functional annotation of the protein means that if one protein function is determined, the proteins linked to this protein may have similar functions. From Figure 3, we can see that A1Y2K1 is involved in the control of cell growth, brain development and mature brain function, plays an important role in the regulation of intracellular calcium levels. A1Y2K1 also plays important roles in the regulation of axon growth, axon guidance, and neurite extension (http://www.uniprot.org/uniprot/A1Y2K1) [40]. In rats, this protein also has these functions. However, in human and mouse, in addition to these functions, A1Y2K1, together with isoform 2, shows a greater ability to mobilize cytoplasmic calcium compared with isoform 1. This protein is involved in 417 interactions, so the 417 interacting proteins may also have similar functions. Using this method, we could infer that A1Y2K1 may be the non-receptor type of tyrosine kinase involved in interleukin-3 and interleukin-23 signal transduction. A1Y2K1 may play a role in leptin signaling and body weight control, because O19064, interacted with A1Y2K1, has these functions. From the annotation of the other proteins, the same conclusions could be drawn.

## 4 Conclusions

Some protein interactions in the cell are transient, and unstable; thus, experiment-based research can hardly capture these interactions. Bioinformatics-based analysis compensates for this shortcoming, with results revealing that the predicted PPI networks are considerably reliable. The current research is an important pioneering work on protein function research.

## 5 Methods

### 5.1 Data resources for PPIs

About 25,767 pig and 105,828 human protein sequences were obtained from the Uniprot (release 2011_05-May 3, 2011) database (http://www.uniprot.org/) [40] and saved in FASTA format. Also, 76,095 mouse and 40,218 rat protein sequences were downloaded from Uniprot (release 2011_10-Oct 19, 2011) database. We then downloaded human protein sequences and human PPIs from the HPRD (release 9) database (http://www.hprd.org/download) [17]; these were saved under the filenames HPRD_Release9_041310.tar.gz and HPRD_FLAT_FILES_041310.tar.gz, respectively. In total, 30,046 protein sequences and 39,240 interactions were obtained. BIOGRID-ALL-3.1.81.tab2.zip, BIOGRID-IDENTIFIERS-3.1.81.tab.zip were downloaded from Biogrid (http://thebiogrid.org/download.php) [20], which reported a large number of interactions, and we extracted mouse and rat PPIs from them. BIND is one of the most comprehensive interaction databases at present. Up to 136,512 interactions and all of the domains were downloaded from the BIND database, including 730 species and viruses. Porcine domains were also downloaded. Moreover, we downloaded Pfam_ls.gz from the Pfam [41] (ftp://ftp.sanger.ac.uk/pub/databases/Pfam/releases/Pfam22.0/) database. Through this file and Hmmer-2.3.2, we generated Pfam domain annotations for proteins in our predicted PPIs. Finally, Pfam domain-interacting pairs [38] in the iPfam [42] database were obtained from ftp://ftp.sanger.ac.uk/pub/databases/Pfam/releases/Pfam21.0/database_files/.

### 5.2 Equipments and softwares

The equipments included computers and servers, which were mainly used to run the prediction and verification program. The softwares employed were BLAST, Cytoscape_v2.8.1, Hmmer-2.3.2 and SPSS (version 17.0). BLAST was used for the Interologs, the visualization tool for the biological networks was Cytoscape [43]. Hmmer-2.3.2 enabled us to create Pfam domain annotations [38] for the input proteins and SPSS was used as a statistical and mapping tool.

### 5.3 Interolog-based prediction of the porcine PPI network

We ran local BLAST using protein sequences of human, mouse and rat with those of pig to obtain the orthologs. The Poisson distribution of Interolog showed that an identity equal to 36% was the cutoff point. To further determine the best cut-off point, we also analyzed other cut-off points (Table 5). Table 1 showed that the accuracy and precision at a cut-off point of 36% were less than those at other cut-off points. Although a 100% cut-off point yielded the highest levels of accuracy and precision, the number of PPIs at this point was particularly small. Finally, 70% was determined to be a relatively good cut-off point and the average query coverage was > 90%, and 20,213 porcine PPIs were successfully predicted, excluding self-interactions.

**Table 5:** PPIs prediction using Interolog method

Interolog	Predicted PPI	Accuracy	Precision
36%	21,192	69.48%	80.27%
70%	20,213	92.20%	92.97%
75%	18,859	93.29%	93.83%
80%	17,166	94.77%	95.07%
85%	14,542	96.20%	96.33%
90%	11,352	97.49%	97.52%
95%	6,496	98.03%	98.04%
100%	569	98.62%	98.62%

### 5.4 D-MIST-based prediction of the porcine PPI network

In D-MIST, a PSSM is necessary to predict porcine PPIs. PSSM is a motif descriptor that attempts to capture the intrinsic variability characteristic of sequence patterns. The PSSM principle ascertains the extent of similarity between some sequences and collected sequences, to construct a scoring matrix.

Score(position,aminoacids)=(q+p)∕(N+B)

Where *q *is the observed count for the amino acids at a given location, *p *is the pseudocount, *N *is the total number of sequences (equal to the maximum number of observed counts), and *B *is the total number of allocated pseudocounts (http://www.people.vcu.edu/~elhaij/IntroBioinf/Scenarios/Scenario5-PSSM.html).

We downloaded PSSMs from *Doron Betel *et al [12], which contained information on the domain and motif interaction, and from which 204 species were derived. We inferred that two proteins interacted if one protein had a domain and another had information of a corresponding motif. We also removed self-interactions and redundancies. In total, 331,484 interactions were predicted based on the PSSMs.

### 5.5 M-MIST-based prediction of the porcine PPI network

Reasons to use M-MIST method are that the result of M-MIST prediction shows a similar accuracy with that of D-MIST prediction, and more importantly, there only exists a small overlap between results of M-MIST and D-MIST prediction. So we used M-MIST to supplement the results of D-MIST to make them more comprehensive. We defined binding motifs as two or more motifs existed in a protein binding a protein at the same time. Each motif was a polypeptide segments of five residues or longer, in which the amino acids side chains were < 4 Å away from the interacting proteins [12]. Two motif residues were segregated by two non-contact residues at most. Furthermore, the motif residues were in direct contact with the interacting protein [12]. M-MIST method was preformed as followings: first, we extracted all protein interactions from BIND, then draw all the binding sites of the proteins and pick up motifs according to the definition the motif. After that, we defined a motif group as all the motifs of a protein. Then we reserved the group in which the number of motifs was equal or more than two. And finally we obtained interactions map between motif groups in the light of PPIs in BIND. Now, this map can be used to predict the protein interaction of pig. If a porcine protein matches with one motif group, and another protein matches with another motif group interacting with the former motif group, then these two proteins can be thought to have interaction, which means two proteins were predicted to interact with each other if they matched the interaction profiles. A total of 11,559 non-redundant PPIs were collected from 730 species and viruses from the BIND database. We excluded self-interactions and constructed interaction profiles. We attempted to predict interactions between all porcine proteins by searching the matching proteins.

### 5.6 Verification of PPIs

Validating the porcine PPI network is difficult, because there exists rarely any swine PPIs at present. Several methods have been proposed for the verification of PPI data [10,44-47]. In this section, we described two effective methods.

Through Hmmer-2.3.2 and Pfam database (Pfam_ls, release 22.0), we constructed Pfam domain annotations for proteins in the predicted PPI networks. The default settings were used to conduct Pfam searching. We retained proteins with e-values less than or equal to 0.01. As a result, many proteins were annotated by the Pfam domain in our predicted PPI network. The number of Pfam domain-annotated protein interactions, as well as PPIs related to the Pfam domain-interacting pairs in the iPfam database (release 21.0) was counted (Table 3). To facilitate comparison, we generated random networks from the 25,767 sequences in the Uniprot database every time using sampling with replacement and the random process was repeated 100 times, then we got the distribution of the number of randomized PPIs related to the Pfam domain-interacting pairs in 100 randomized networks. Furthermore this distribution was used to determine statistical significance of our results. Finally, we evaluated the reliability of our predicted networks by comparing the number of PPIs related to the Pfam domain-interacting pairs between the predicted and randomized networks.

Using the Gene Ontology Annotation is another method to verify predicted swine PPIs. The recently released GO annotations of pig were downloaded from http://www.ebi.ac.uk/QuickGO/[48]. The GO terms were organized according to three independent hierarchies: Biological Process, Molecular Function, and Cellular Component [49]. Since a pair of interacting proteins generally have related but not identical functions, they should have some but not all of their GO annotations in common. Therefore, we considered that the two proteins interacting with each other if they shared at least one GO term in any of the three GO categories, and we calculated the percentage of the predicted PPIs sharing GO terms [38]. For comparison, we randomly chose 10,000 pairs of porcine protein from all pig proteins every time using sampling with replacement, and we preformed this for 10,000 times altogether. To evaluate the network, we compared the proportion of the protein pairs sharing at least one GO term in any of the three GO categories in the predicted and 10,000 randomized networks. Then, we evaluated the reliability by comparing the percentage of PPI pairs sharing GO terms in the predicted PPI network and 10,000 randomized networks.

Accuracy and precision were the statistical measures of the tests. Based on the evaluation, a positive and a negative set were selected, and then used to assess the results mentioned above. 2,732 pairs of chimpanzee PPI data with high confidence were selected from STRING database, all these 2,732 pairs should be the result of experimental verification and that their "combined score" > 950 which were used as a gold standard positive set (GSPs) [14]. A golden standard negative set (GSNs) of 3,000 protein pairs was defined, in which proteins were randomly selected from Uniprot. We used PPIs reconstructed from the GSPs and GSNs by the three methods to analyze the accuracy and precision of the predicted results. We supposed that a positive prediction was right if it was included in our golden standard positive (GSP) set and that a negative prediction was right if it was included in our golden standard negative (GSN) set because we cannot always guarantee that a prediction was right [7]. Accuracy was calculated by TP+TN/(TP+TN+FN+FP), and was a part of correct predictions. True positive (TP) was defined as the number of correctly predicted PPIs, while false positive (FP) was defined as the number of non-PPIs predicted as PPIs. True negative (TN) was defined as the number of correctly predicted non-PPIs, and false negative (FN) was defined as the number of PPIs predicted as non-PPIs. For PPIs, precision, the percentage of the PPIs correctly predicted among all the predictions, was calculated by TP/(TP+FP). For non-PPIs, precision was calculated by TN/(TN+FN). Therefore, the precision of the tests was obtained from the average of two precision values (for PPIs and non-PPIs).

## Abbreviations

PPI: protein-protein interaction; D-MIST: domain-motif interactions from structural topology; M-MIST: motif-motif interactions from structural topology; PSSMs: position-specific scoring matrices; DIP: Database of Interacting Proteins; HPRD: Human Protein Reference Database; MINT: the Molecular INTeraction database; BIND: Biomolecular Interaction Network Database; Biogrid: Biological General Repository for Interaction Datasets; MIPS: The MIPS Mammalian Protein-Protein Interaction Database; GSP: golden-standard positive; GSN: golden-standard negative; TP: true positives; TN: true negatives; FP: false positives; FN: false negatives

## Competing interests

The authors declare that they have no competing interests.

## Authors' contributions

FW, ML and BXS significantly contributed to the present research by designing the conducting the experiment, assembling and verifying the datasets for analysis, and writing the initial draft of the manuscript. DYL supervised the statistical procedures and analysis of the results. HMP, YG, JFH, DLZ revised the manuscript. All authors read and approved the final manuscript.

## Supplementary Material

Additional file 1 **Statistical analysis of the degrees of nodes**. Click here for file

Additional file 2 **The degrees of nodes**. Click here for file

Additional file 3 **The overlap between the predicted interactions and domain family pairs from iPfam**. Click here for file

Additional file 4 **The distribution of the 10,000 randomized networks sharing GO terms**. Click here for file
