# Supplementary figures and images for: Prediction and characterization of protein-protein interaction networks in swine
Source: Proteome Sci. 2012 Jan 10;10:2. doi: 10.1186/1477-5956-10-2 (PMC3306829; doi:10.1186/1477-5956-10-2)

## The distribution for random predictions

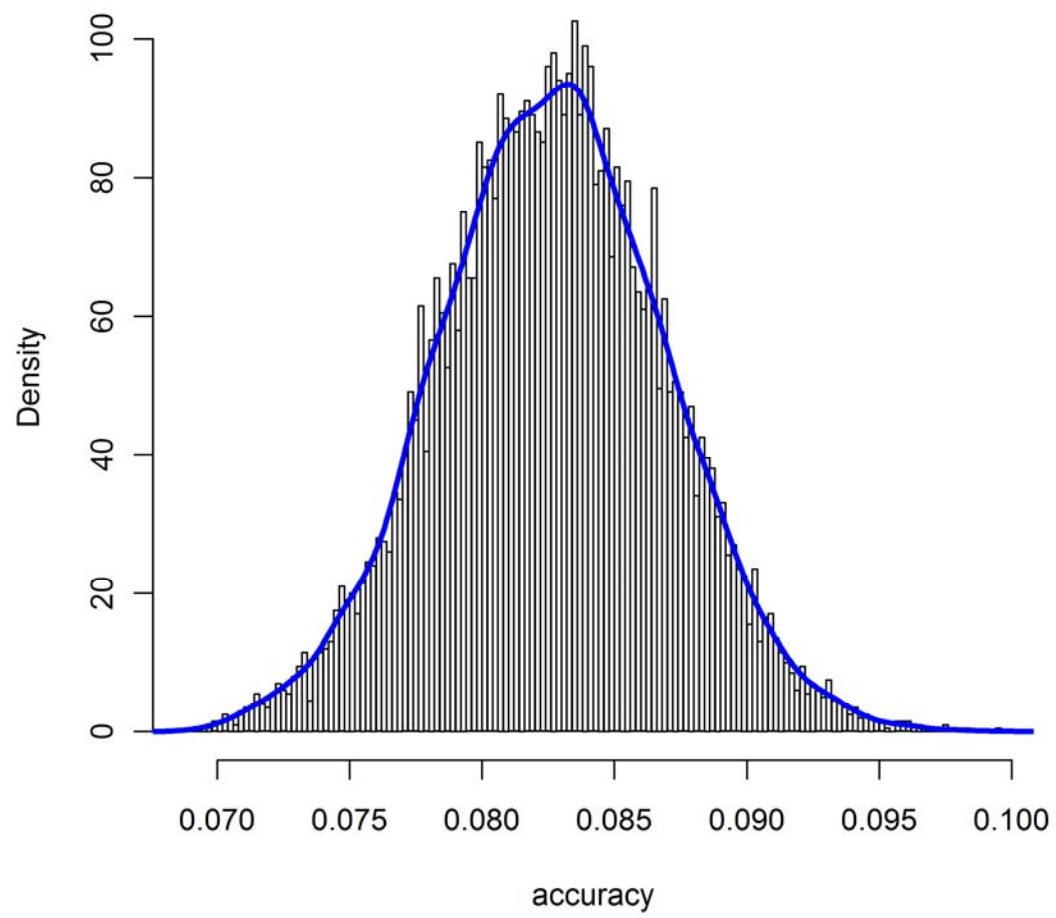

Supplement: Additional file 4 — The distribution of the 10,000 randomized networks sharing GO terms. [file 1477-5956-10-2-S4.PDF]
